# COVID-19 in patients with rheumatological diseases in the Eastern Province of Saudi Arabia

**DOI:** 10.25122/jml-2023-0037

**Published:** 2023-06

**Authors:** Safi Alqatari, Ameera Nemer, Manal Hasan, Raed Bukhari, Reem Al Argan, Dania Al Khafaji, Abrar Alwaheed, Alaa Alzaki, Marwan Al-wazza, Sara Al Warthan, Abir Al Saeed, Feda Albeladi, Hashim Almeer, Reem AlSulaiman, Ahmed Abu Quren

**Affiliations:** 1 Department of Internal Medicine, College of Medicine, King Fahd Hospital, Imam Abdulrahman Bin Faisal University, Khobar, Saudi Arabia

**Keywords:** COVID-19 infection, rheumatological disease, disease severity, disease outcome, mortality, COVID-19: Coronavirus disease 2019, SARS-CoV-2: Severe Acute Respiratory Syndrome Coronavirus 2, DM: Diabetes mellitus, ICU: Intensive Care Unit, LDH: Lactate dehydrogenase, ESR: Erythrocyte sedimentation rate, CRP: C-reactive protein, CK: Creatine kinase, ANA: Antinuclear antibody, ARDS: Acute respiratory distress syndrome, IQR: Interquartile range, ORs: Odds ratios, CI: Confidence intervals, SD: Standard deviations, AOR: Adjusted odds ratio, CKD: Chronic kidney disease, GI: Gastrointestinal, WHO: World Health Organization, SLEDAI: Systemic Lupus Erythematosus Disease Activity Index, SLE: Systemic lupus erythematosus, RA: Rheumatoid arthritis, CTD: Connective tissue disease, GPA: Granulomatosis with polyangiitis, PAN: Polyarteritis nodosa, PsA: Psoriatic arthritis, HCQ: Hydroxychloroquine, MTX: Methotrexate, AZA: Azathioprine, MMF: Mycophenolate mofetil, TNF inhibitor: Tumor necrosis factor inhibitor, IFN: Interferon, BMI: Body mass index, CT: Computed tomography scan, DMARDs: Disease-modifying antirheumatic drugs, IL: Interleukin, SpO2: Oxygen saturation, LOH: Length of hospitalization, AT2: Type 2 alveolar cells, ACE2: Angiotensin-converting enzyme 2, COPD: Chronic obstructive pulmonary disease, ILD: Interstitial lung disease

## Abstract

The severity of the 2019 coronavirus disease (COVID-19) and its effects remain unpredictable. Certain factors, such as obesity, hypertension, and type 2 diabetes mellitus, may increase the severity of the disease. Rheumatology experts suggest that patients with active autoimmune conditions and controlled autoimmune diseases on immunosuppressive therapy may be at higher risk of developing severe COVID-19. In this retrospective observational study, we aimed to examine the patterns of COVID-19 in patients with underlying rheumatological diseases and their association with disease severity and hospital outcomes. A total of 34 patients with underlying rheumatological diseases who tested positive for severe acute respiratory syndrome coronavirus-2 (SARS-CoV-2) by polymerase chain reaction (PCR) were included between March 2020 and April 2021 at King Fahd Hospital of the University. The study population consisted of 76.47% female and 23.53% male patients, with a mean age ranging from 20 to 40 years. Female gender (p=0.0001) and younger age (p=0.004) were associated with milder disease. The most frequent rheumatological disease was systemic lupus erythematosus (SLE) (38.24%), which was associated with a milder infection (p=0.045). Patients treated with mycophenolate mofetil (MMF) had a milder disease course (p=0.0037). Hypertension was significantly associated with severe COVID-19 disease (p=0.037). There was no significant relationship between SLE and the need for ICU admission. Patients on hydroxychloroquine and MMF tended to develop milder disease, and there was no association between the severity of the infection and the treatment with steroids.

## INTRODUCTION

In March 2020, the World Health Organization (WHO) declared SARS-CoV-2 a pandemic that can spread primarily by inhalation of droplets produced by an infected person. There are more than 20 million confirmed cases worldwide [1]. Elderly patients, those with comorbidities such as diabetes mellitus, cardiovascular diseases, chronic lung diseases, cancer, rheumatological and immunological diseases, and those on immunosuppressive therapy are considered at increased risk of SARS-CoV-2 infection. Among these high-risk groups, patients with rheumatological diseases, in particular, are of special concern due to the similarity between the pathogenesis of SARS-CoV-2 and rheumatological diseases and the effects of immunosuppressive therapy [2, 3].

Most rheumatic patients suffering from SARS-CoV-2 present fever as the first symptom, with cough, sputum, and dyspnea being the most common respiratory symptoms. The clinical characteristics of SARS CoV-2 can mimic the flare-up of rheumatic diseases, such as high-grade fever, arthralgia, myalgia, and fatigability, in addition to lymphopenia, a high erythrocyte sedimentation rate (ESR), C-reactive protein (CRP), and creatine kinase (CK). Some studies reported a high D-dimer level, positive antinuclear antibodies (ANA), and antiphospholipid antibodies in patients infected with SARS-CoV-2. Ground glass opacity is the most common finding in chest computed tomography scans (CT) of rheumatic patients suffering from SARS-CoV-2 infection but can be confused with interstitial lung disease related to connective tissue diseases. However, it can be differentiated by the persistence of fibrotic lung changes after the resolution of the ground glass opacity following the recovery of infection, which favors the diagnosis of interstitial lung disease [4]. Moreover, lymphocyte infiltration of various organs, including but not limited to the lung, was reported in the autopsy of SARS CoV-2 patients, a common finding in rheumatic diseases [5].

While some studies reported a more severe form of SARS-CoV-2 infection in patients receiving high-dose glucocorticoids and disease-modifying antirheumatic drugs (DMARDs), other studies pointed towards promising outcomes with the use of IL-1 and IL-6 inhibitors, which help mitigate the immunological process that mediates more severe infections [6]. Treatment with tocilizumab, an IL-6 inhibitor, was associated with a milder disease course [7], whereas methotrexate and rituximab were associated with a higher risk of hospitalization, and high-dose steroid treatment (10 mg/day) was found to increase mortality [8,9].

Upon reviewing the literature, we found that the risk of SARS CoV-2 infection was higher in elderly patients with comorbidities than in patients with rheumatological diseases or those using immunosuppressive therapy, indicating that continuation of their regimen is crucial to avoid rheumatological disease flares [10, 11]. Mortality rates and risk of hospitalization in patients with rheumatological diseases were similar to those in the general population. However, there was an increased risk of critical care admission and the need for mechanical ventilation [12, 13].

Given the limited knowledge available on the behavior of COVID-19 in patients with rheumatological diseases in the Gulf area, there is a crucial need to expand our understanding of this field. Previous studies conducted in Saudi Arabia and Egypt have provided valuable insights into the clinical characteristics and outcomes of COVID-19 in patients with rheumatic diseases, particularly emphasizing the impact of age as a predictor of severe infection [14-16]. However, the specific distinctions and implications of COVID-19 in the context of rheumatological diseases in the Gulf region remain largely unexplored.

Therefore, this study aimed to address this knowledge gap by investigating the patterns and implications of COVID-19 in patients with rheumatological diseases in the Gulf area.

## MATERIAL AND METHODS

We conducted a retrospective observational study to investigate the pattern of COVID-19 infection in patients with underlying rheumatological diseases. The study was conducted at King Fahd Hospital of the University in Al-Khobar, Eastern Province of Saudi Arabia. We reviewed the medical records of patients who tested positive for the SARS-CoV-2 PCR test between March 2020 and April 2021 and had a documented rheumatological disease. We included all patients 18 years with a positive COVID-19 PCR test. Patients with incomplete medical records, pregnancy, malignancy, and immunodeficiency syndromes, including human immune deficiency infection, were excluded from the study to minimize confounding effects

### Data collection

Data collection was performed by carefully reviewing the hospital charts and electronic health records of the patients. Information was collected in the following six sections:

1. Demographics: age, gender, and nationality
2. Underlying rheumatological disease and organ involvement
3. Other comorbid conditions
4. Medications received by the patients
5. COVID-19 symptoms and severity: Symptoms were graded based on the World Health Organization (WHO) guideline, categorizing disease severity as mild (respiratory symptoms but no evidence of viral pneumonia or hypoxia), moderate (clinical signs of pneumonia: fever, cough, dyspnea, but oxygen saturation 90% on room air), severe (clinical signs of pneumonia plus one of the following: respiratory rate > 30 breaths/min; or SpO2 90% on room air) and critical (which includes ARDS, sepsis or septic shock or acute thrombosis or acute thrombosis or acute thrombosis)
6. COVID-19 outcome: The outcome was determined based on the length of hospitalization (LOH), the need for intensive care unit (ICU) admission, mechanical ventilation, and death.

### Data analysis

Statistical analysis was performed using SAS version 9.2 (SAS Institute, Inc., Cary, NC) and R (R Foundation for Statistical Computing, Vienna, Austria). Continuous data were summarized as means and standard deviations (SD) or medians and interquartile ranges (IQR), while categorical data were summarized as numbers or percentages. Statistical comparisons between groups were conducted using the Chi-square or Fisher's exact test for categorical variables and the student t-test or Mann–Whitney U test for continuous data. A multivariate regression model was used to identify risk factors predictive of the study outcomes. Adjusted odds ratios (AOR) with 95% confidence intervals (CI) and p-values were reported for the regression analysis. The level of statistical significance was set at p<0.05.

## RESULTS

### Patient demographics

The analysis includes a total of 34 patients. Of the 34 patients, 26 (76.47%) were females, and 32 (94.12%) were of Saudi nationality. Most patients (47.06%) fell into the age range of 41-60 years, while only 8.82% were older than 60 years.

### Rheumatological diseases and treatment

**Figure 1:**
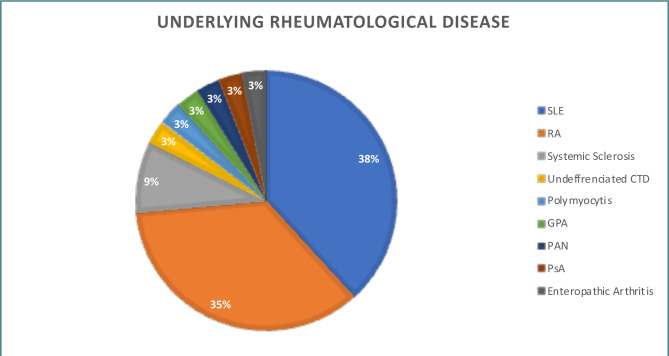
Underlying rheumatological disease

Figure 1 and Table 1 show the distribution of rheumatological diseases among the patients. Systemic lupus erythematosus (SLE) was the most common underlying rheumatological disease, accounting for 38% of cases, followed by rheumatoid arthritis (RA) at 35%. Other rheumatological diseases were less prevalent. Joints were the most commonly involved organs (64.71%) in patients with rheumatological diseases, followed by the lung and kidneys (8.82%). Most patients (64.71%) received treatment with steroids for their rheumatological diseases, with 22.73% receiving a dose greater than 10mg per day. Methotrexate, mycophenolate mofetil (MMF), and rituximab were the other commonly used medications, accounting for 26.47%, 23.53%, and 20.59% of cases, respectively.

**Table 1:** Column I. Patient Characteristics

Category		Number	Percentage (%)
Age	20-40 Years	15	44.12
	41-60 Years	16	47.06
	>60 Years	3	8.82
Gender	Female	26	76.47
	Male	8	23.53
Nationality	Saudi	32	94.12
	Non-Saudi	2	5.88
Rheumatological Disease	SLE	13	38.24
	RA	12	35.29
	Systemic Sclerosis	3	8.82
	Undifferentiated CTD	1	2.94
	Polymyositis	1	2.94
	GPA	1	2.94
	PAN	1	2.94
	PsA	1	2.94
	Enteropathic Arthritis	1	2.94
Major organ involved	Joints	22	64.71
	Heart	2	5.88
	Kidney	3	8.82
	Lung	3	8.82
	Blood	2	5.88
	Bowel	1	2.94
	Sinus	1	2.94
Medications	Steroid	22	
	- <10 mg/d	17	64.71
	- >10 mg/d	5	77.27
	HCQ	6	22.73
	HCQ + MXT	2	17.65
	HCQ + AZA	1	5.88
	HCQ + MMF	4	2.94
	AZA	2	11.76
	Methotrexate	9	5.88
	MMF	8	26.47
	HCQ+MMF	4	2.94

**Table 1:** Column II. Patient Characteristics

Category		Number	Percentage (%)
Medications	AZA	2	11.76
	Methotrexate	9	5.88
	Biologics		
	- TNF inhibitor	6	17.65
	- Abatacept	1	2.94
	- Rituximab	7	20.59
Comorbidities	Diabetes	8	23.53
	Hypertension	11	32.35
	Obesity		
	· BMI (18.5-24.9)	13	38.24
	· BMI (25-29.9)	7	20.59
	· BMI (>=30)	14	41.18
	Smoking	3	8.82
	Heart disease	4	11.76
	CKD	2	5.88
COVID-19 symptoms	Fever	14	41.18
	Cough	12	35.29
	Dyspnea	11	32.35
	Anosmia	4	11.76
	GIT	8	23.53
	Myalgia	9	26.47
	Fatigue	9	26.47
	Headache	7	20.59
	Sore throat	2	5.88
COVID-19 severity	Mild	14	41.18
	Moderate	11	32.35
	Severe	9	26.47
Admission	Home treatment	14	41.18
	Inpatient	20	58.82
Outcome	Hospital stay (> 7 days)	11	29.4
	ICU	9	26.47
	Mechanical Ventilation	5	14.71
	Death	0	0

Abbreviations: SLE, Systemic lupus erythematosus; RA, Rheumatoid arthritis; CTD, connective tissue disease; GPA, Granulomatosis with polyangiitis; PAN, Polyarteritis nodosa; PsA, Psoriatic arthritis; HCQ, Hydroxychloroquine; MXT, Methotrexate; AZA, azathioprine; MMF, Mycophenolate mofetil; TNF inhibitor, tumor necrosis factor inhibitor; BMI, body mass index; CKD, chronic kidney disease; GIT, gastrointestinal tract; ICU, intensive care unit

### Other comorbidities

Hypertension was the most frequent comorbidity observed in the study population (32.35%), followed by obesity (41.18%) and diabetes (23.53%).

### Clinical manifestations of COVID-19 disease

The most common symptoms observed in patients with COVID-19 were fever (41.18%), followed by respiratory symptoms in the form of cough (35.29%), dyspnea (32.35%), and headache (20.59%). Other symptoms, such as anosmia (11.76%) and sore throat (5.88%), were also observed in a minority of patients.

### Severity of COVID-19 disease

14 patients (41.18%) had a mild COVID-19 infection and were managed as outpatients, while 20 (58.82%) required hospital admission. Within the hospitalized group, 9 patients (26.47%) had severe COVID-19 infection and required admission to a critical care area, and 5 (14.71%) developed severe respiratory distress syndrome and required mechanical ventilation. The remaining 11 patients (32.35%) were hospitalized on the regular medical floor for an average of 7 days. Fortunately, no mortality was reported in the study (Table 1).

### Association between COVID-19 disease severity and outcome with rheumatological disease

Univariate analysis revealed that patients with SLE tended to develop a milder course of COVID-19 disease than others (p=0.045). The multivariate analysis showed that patients with rheumatoid arthritis tended to have a more critical COVID-19 course than SLE patients, although not statistically significant. We could not calculate the p-value for the rest of the rheumatological diseases because of the small number of patients. The analysis of the outcome of COVID-19 disease in relation to rheumatological diseases did not show a statistically significant association (Table 2).

**Table 2:** Severity in relation to age, gender, comorbidities, rheumatologic and background lung disease (multivariate analysis)

	Severe/Critical OR (95% CI)	p-values
Gender	0.193 (0.03 - 1.3)	0.083
Age	2.368 (0.6 - 8.7)	0.196
BMI	1.03 (0.9 - 1.13)	0.503
Background lung disease	0.274 (0.01 - 8.7)	0.463
Diabetes	0.328 (0.013 - 8.1)	0.496
Hypertension	27.3 (1.54 - 484.9)	0.024
Smoking	1.11 (0.02 - 67.1)	0.961
Heart disease	13.5 (0.55 - 336.1)	0.112
SLE	0.722 (0.55 - 336.1)	0.70

Abbreviations: BMI, body mass index; SLE, Systemic lupus erythematosus

Even though SLE was the most common rheumatological disease observed in our study, the multivariate analysis showed no significant relationship between SLE and the need for intensive care unit (ICU) admission or hospitalization (95% CI 0.1–23.9; p=0.9 and 95% CI 0.2–48.8; p=0.47, respectively) (Table 3).

**Table 3:** Relationship between age, gender and comorbidities with severity of COVID-19 (n=34)

Category		Total Number	Severity			p-value
			Mild Number (Percentage)	Mod/Severe Number (Percentage)	Critical Number (Percentage)	
Age	20-40	15	11 (73.33%)	3 (20%)	1 (6.66%)	0.004
	41-60	16	10 (62.5%)	0	6 (37.5%)	0.16
	>60	3	1 (33.33%)	0	2 (66.66%)	0.45
Sex	Female	26	19 (73.07%)	2 (7.69%)	5 (19.23%)	0.0001
	Male	8	3 (37.5%)	1 (12.5%)	4 (50%)	0.117
Comorbidities	Diabetes	8	3 (37.5%)	0	5 (72.5%)	0.17
	Hypertension	11	3 (27.27%)	0	8 (72.72%)	0.037
	Obesity					
	· BMI (18.5-24.9)	13	13 (100%)	0	0	NA
	· BMI (25-29.9)	7	2 (28.57%)	2 (28.57%)	3 (42.85%)	0.60
	· BMI (>=30)	14	7 (50%)	1 (7.14%)	6 (42.85%)	0.013
	Smoking	3	1 (33.33%)	0	2 (66.66%)	0.455
	Heart disease	4	1 (25%)	1 (25%)	2 (50%)	0.50
	CKD	2	0	0	2 (100%)	NA

Abbreviations: BMI, body mass index; CKD, chronic kidney disease

### The relationship between COVID-19 severity and outcome and rheumatological diseases and medications

Patients receiving hydroxychloroquine had a milder course of COVID-19, and none of them required critical care admission (p=0.027). There was no significant association between the severity of COVID-19 infection and treatment with steroids, regardless of the dose. However, of the 8 patients treated with mycophenolate mofetil (MMF), only one required ICU admission, suggesting a milder disease course (p=0.0037). There was no significant association between treatment with rituximab and disease severity (Table 4).

**Table 4:** Relationship between underlying rheumatologic disease, background lung disease and medications with COVID-19 severity

Category		Total Number	Severity			p-value
			Mild Number (Percentage)	Mod/Severe Number (Percentage)	Critical Number (Percentage)	
Underlying Rheumatic Disease	SLE	13	9 (69.23%)	2 (15.38%)	2 (15.38%)	0.045
	RA	12	8 (66.66%)	0	4 (33.33%)	0.109
	Systemic Sclerosis	3	3 (100%)	0	0	NA
	Undiff. CTD	1	0	1 (100%)	0	NA
	GPA	1	0	0	1 (100%)	NA
	PAN	1	1 (100%)	0	0	Na
	PsA	1	0	0	1 (100%)	NA
	Enterop. Arthritis	1	1 (100%)	0	0	NA
	Polymyositis	1	0	0	1 (100%)	NA
Lung Disease		3	2 (66.66%)	0	1 (33.33%)	
Medications	Steroid	22				
	- <10 mg/d	17	10 (58.82%)	2 (11.76%)	5 (29.41%)	0.455
	- >10 mg/d	5	4 (80%)	0	1 (20%)	0.079
	HCQ	6	5 (83.33%)	1 (16.66%)	0	0.027
	HCQ + MXT	2	1 (50%)	0	1 (50%)	1
	HCQ + AZA	1	0	1 (100%)	0	NA
	HCQ + MMF	4	2 (50%)	1 (25%)	1 (25%)	0.50
	AZA	2	2 (100%)	0	0	NA
	Methotrexate	9	6 (66.66%)	0	3 (33.33%)	0.168
	MMF	8	7 (87.5%)	0	1 (12.5%)	0.0037
	Biologics					
	- TNF inhib	6	4 (66.66%)	0	2 (33.33%)	0.269
	- Abatacept	1	1 (100%)	0	0	NA
	- Rituximab	7	3 (42.85%)	1 (14.28%)	3 (42.85%)	0.25

Abbreviations: SLE, Systemic lupus erythematosus; RA, Rheumatoid arthritis; CTD, connective tissue disease; GPA, Granulomatosis with polyangiitis; PAN, Polyarteritis nodosa; PsA, Psoriatic arthritis; HCQ, Hydroxychloroquine; MXT, Methotrexate; AZA, azathioprine; MMF, Mycophenolate mofetil; TNF inhibitor, tumor necrosis factor inhibitor

### The relationship between COVID-19 severity and outcome with comorbidities

Age was significantly associated with disease severity, with patients aged 20-40 years experiencing a milder course of COVID-19 (p=0.004). Female patients had a milder disease course than male patients (p=0.0001). Hypertension was significantly associated with severe COVID-19 disease in 72.72% of patients (p=0.037), and a significant proportion (42.85%) of hypertensive patients also had a BMI of > 30 kg/m2 (p=0.013). No significant associations were found between diabetes or smoking and the severity of COVID-19 infection (Table 5). Moreover, a multivariate logistic regression analysis showed that hypertension was an independent predictor of COVID-19 disease severity (95% CI 1.54–484.9; p=0.024) and ICU admission (95% CI 2.92–1515.14; p=0.009). Older age was also an independent predictor of severe or critical COVID-19 disease (95% CI 1.62–73.49; p=0.014). In addition, males were significantly associated with a higher risk of critical care admission than females (95% CI 0.01–0.91; p=0.014). However, patients with SLE had no significant relationship with the need for ICU admission (95% CI 0.55–336.1; p=0.70) (Table 6).

**Table 5:** Relationship between underlying rheumatologic disease, background lung disease and medications with COVID-19 outcomes

Variable		Number	Hospital stay	p-value	ICU admission Number (%)	p-value	Mechanical ventilation Number (%)	p-value
Underlying Rheumatic Disease	SLE	13	3 (23.1%)	0.58	2 (15.38%)	0.312	0	0.29
	RA	12	4 (33.3%)		4 (33.33%)		1 (8.33%)	
	Others	9	3 (33.3%)		3 (33.3%)		1 (11.1%)	
Lung Disease		3			1 (33.33%)		0	
Medications	Steroid	22		0.53		0.68		0.58
	- <10 mg/d	17	6 (35.3%)		5 (29.41%)		1 (5.88%)	
	- >10 mg/d	5	1 (20%)		1 (20%)		0	
	HCQ	6			0		0	
	HCQ + MXT	2			1 (50%)		0	
	HCQ + AZA	1			0		0	
	HCQ + MMF	4			1 (25%)		0	
	AZA	2			0		0	
	Methotrexate	9			3 (33.33%)		1 (11.11%)	
	MMF	8			1		0	
	Biologics							
	- TNF inhib	6	0		2 (33.33%)		1 (16.66%)	
	- Abatacept	1	0		0		0	
	- Rituximab	6	3 (50%)		3 (42.85%)		0	

Abbreviations: SLE, Systemic lupus erythematosus; RA, Rheumatoid arthritis; HCQ, Hydroxychloroquine; MXT, Methotrexate; AZA, azathioprine; MMF, Mycophenolate mofetil; TNF inhibitor, tumor necrosis factor inhibitor, ICU, intensive care unit

**Table 6:** The outcome in relation to age, gender, comorbidities, rheumatologic and background lung disease (multivariate analysis)

	Hospital stay (> 7 days) OR (95% CI)	p-values	ICU OR (95% CI)	p-values
Gender	0.05 (0.01 - 0.5)	0.013	0.09 (0.01 - 0.91)	0.041
Age	7.9 (1.3 - 47.2)	0.023	10.9 (1.62 - 73.49)	0.014
BMI	1 (0.9 - 1.1)	0.873	0.99 (0.89 - 1.11)	0.868
Background lung disease	0.4 (0 - 19.5)	0.65	1.75 (0.03 - 90.72)	0.782
Diabetes	0.8 (0 - 22.3)	0.9	0.88 (0.04 - 20.28)	0.937
Hypertension	80.6 (3.2 - 2062.7)	0.008	66.5 (2.9 - 1515.1)	0.009
Smoking	2.7 (0 - 192.7)	0.645	3.91 (0.03 - 497.2)	0.581
Heart disease	41.2 (0.8 - 2217.7)	0.068	3.6 (0.12 - 105.21)	0.461
SLE	2.8 (0.2 - 48.8)	0.47	1.2 (0.1 – 23.9)	0.9

Abbreviations: BMI, body mass index; SLE, Systemic lupus erythematosus; ICU, intensive care unit

## DISCUSSION

This study addressed the knowledge gaps by evaluating the clinical characteristics of COVID-19 infection in patients with rheumatological disorders, specifically in the Eastern Province of Saudi Arabia, where data is scarce. It is the first single-center study conducted in this region and one of the few studies in Saudi Arabia that investigates the pattern of COVID-19 infection in patients with rheumatic diseases.

In our study, 94.1% of our subjects were Saudi citizens, which is compatible with the Saudi study published by Hassen *et al*., where most participants were Saudi (84.8%) compared to 15.1% non-Saudi [17]. Females accounted for 76.47% of participants, which may be related to the higher prevalence of rheumatological disorders among females compared to males. Many published studies have concluded that females have lower disease severity compared to the male population [18,19]. A higher estradiol level in females may play an important role as a protective factor. Females tend to have a milder clinical course than male patients, as demonstrated in one article by Ramrez-de-Arellano *et al*. about the effects of estradiol on the immune response to COVID-19 infection [19]. The severity of COVID-19 infection in the general population was lower in females as well, as supported by other studies [19,20]. Vahidy *et al*. reported that men are more likely to have COVID-19-related complications such as hypoxemia and acute respiratory distress syndrome (ARDS), a higher rate of ICU admission, and a need for mechanical ventilation.

Moreover, the mortality rate was higher in males (11.6%) compared to females (8.3%) [21]. A similar result was demonstrated in our study, where females were found to have a milder disease course compared to male patients. Our data showed a significant association between male gender and the risk of critical care admission.

It is well known that older patients have a higher disease severity and a higher mortality rate than younger patients [22]. Advanced age is considered one of the most important risk factors for disease severity and, consequently, mortality [23]. Fortunately, there were no deaths in our study. The majority of our patients were between 41 and 60 years of age (47.06%), and 44.12% were younger patients under 40 years. Only one patient under 40 years (6.66%) required critical care admission compared to six patients (37.5%) aged between 41 and 60 years. Our results were compared with previously published data in which older age was associated with severe or critical COVID-19 disease [22, 23].

Impaired innate and humoral immune responses correlate with an increased risk of infection in patients with rheumatological diseases. T cell depletion and excessive production of cytokines, such as IL-1, IL-2, IL-6, IL-7, IL-10, G-CSF, and TNF-α levels, are associated with a more severe form of the disease, making the host immune response crucial for the resolution of SARS-CoV-2 infection [24]. In a review by Fernandez-Ruiz *et al*., it was highlighted that patients with rheumatological diseases, particularly SLE, may have an impaired cellular immune response, hypocomplementemia, and low levels of immunoglobulin, making them more susceptible to COVID-19 infection [18]. In the two studies conducted in Saudi Arabia, the most common rheumatological disease observed was RA (42.7%), followed by SLE (31.9%) [14,17]. The most common rheumatological disease in our study was SLE (38.24%). By applying the Systemic Lupus Erythematosus Disease Activity Index (SLEDAI), 4 patients were in remission, and the other 9 patients had mild to severe disease activity at the time of infection. Rheumatoid arthritis was the second most common disease (32.29%), while other rheumatologic conditions accounted for 29.41% of cases. Only 2 cases of vasculitis were found in our subjects, namely, Polyarteritis Nodosa (PAN) and granulomatosis with polyangiitis. Joints were the most common organ involved (64.71%) in patients with rheumatological diseases, followed by the lung and kidneys (8.82%). Other organs involved were the heart, blood, and gastrointestinal tract [4].

In a recent systematic review and meta-analysis, hypertension was identified as the most common comorbidity in patients with COVID-19, followed by diabetes and obesity. Chronic kidney disease was the most common disease associated with mortality [24, 25]. In our study, we observed a different prevalence of comorbidities. The majority of patients had obesity (41.18%), followed by hypertension (32.35%) and diabetes mellitus (23.5%). Only two patients had chronic kidney disease, and both developed severe COVID-19 infections, consistent with other findings [24]. Of the 9 patients who developed severe COVID-19, 8 were hypertensive (88.88%), 5 were diabetic (55.55%), and 2 were smokers. All patients with a severe COVID-19 infection were overweight (33.33%) or obese (66.66%). Hypertension was significantly associated with severe COVID-19 disease. Notably, 42.85% of hypertensive patients also had a BMI of > 30 kg/m2 and required admission to the critical area. Our data showed no significant association between diabetes and smoking and the severity of the COVID-19 infection.

Regarding the clinical manifestations of COVID-19, Grainger *et al*. found in one study that COVID-19 symptoms were similar between patients with and without rheumatological disorders [26]. Given that the symptoms of COVID-19 infection are similar to those of a disease flare-up, it is difficult to distinguish between the two, especially since both can present elevated erythrocyte sedimentation rate (ESR), C-reactive protein (CRP) and leukopenia. The most common symptoms in those patients are fever and shortness of breath [1]. Our study showed a similar clinical pattern, as the main clinical presentation in our subjects was fever (41.18%), followed by respiratory symptoms in the form of cough (35.29%), dyspnea (32.35%), and headache (20.59%). Other symptoms, such as anosmia (11.76%) and sore throat (5.88%), were also observed in a minority of patients.

Regarding the severity of the COVID-19 disease, WHO classified it into three categories: mild, moderate, and severe. Severe COVID-19 infection is defined by the presence of severe respiratory distress in the form of a respiratory rate of more than 30 breaths/min or an oxygen saturation of less than 90% on room air, the development of sepsis, or ARDS [27]. Generally, multiple risk factors affect the development of severe COVID-19 infection, including advanced age, male gender, comorbidities including underlying lung or cardiovascular diseases, smoking, obesity, and steroid or immunosuppressive medications [28]. In our study, most patients (64.71%) had a milder course of infection. A total of 20 patients (58.82%) required hospitalization, of whom 8.82% and 26.5% had moderate and severe diseases, respectively. Fortunately, there was no reported mortality in our study.

Based on the literature, the need for hospital admission was higher in rheumatological patients compared to the general population. Most of these studies were published at the beginning of the pandemic when most patients with advanced comorbidities and immunological or rheumatological diseases were admitted for close observation, even if they had a mild infection [29]. More recent studies suggest that the prevalence of COVID-19 infection in patients with rheumatological disease is comparable to that in the general population. Nevertheless, the rate of ICU admission and the need for mechanical ventilation was higher in patients with rheumatological disease than in non-rheumatological patients [30]. In our study, of the 34 patients who tested positive for COVID-19, 22 (64.71%) had a mild disease course and did not require hospitalization. However, 12 patients needed hospitalization, and 9 of those (26.47%) had a severe infection and required ICU admission. Only 2 patients (5.88%) required intubation and mechanical ventilation. Participants who developed a severe infection had higher levels of ferritin, D-dimer, and LDH than those with milder disease. The correlation of ferritin and other markers to disease severity is well known in the literature, and it is related to the hyperinflammatory status, which predicts disease severity and clinical outcome [31].

A study published in April 2021 concluded that patients with chronic obstructive pulmonary disease (COPD) or interstitial lung disease (ILD) have a higher risk of developing more severe infections compared to the general population. In contrast to patients with bronchial asthma, the study did not show a strong association with increased severity of COVID-19 infection. Furthermore, compared to the general population, patients with underlying chronic lung disease have a higher rate of gene expression by type 2 alveolar cells (AT2) and angiotensin-converting enzyme type 2 (ACE2) receptors, which results in increased viral replication and more severe infection [32]. This is supported by a Korean cohort study, which shows that patients with ILD have a higher risk for ICU admission, mechanical ventilation, and mortality. However, this study also showed that rheumatological diseases in patients with ILD were not associated with severe COVID-19 infection. This could be attributed to the small number of patients enrolled in the study [33]. Another study investigated the impact of pre-existing lung involvement in patients with a rheumatological disease on the severity of the COVID-19 infection. The study showed that patients with rheumatological diseases with underlying lung involvement had a higher mortality rate (37%) compared with patients with no underlying lung pathology (7.9%) [34].

In our study, only one of three patients with an underlying lung disease developed a severe infection and required ICU admission. He was known to have SLE complicated by pneumonitis and bronchiectasis. The second patient with bronchiectasis in the background of rheumatoid arthritis required hospitalization. The third patient was known to have diffuse systemic sclerosis with non-specific interstitial pneumonia (NSIP) and had mild COVID-19 but did not require hospitalization. We could not compare our results to the literature because we had a small cohort of patients with underlying lung conditions.

Another risk factor contributing to prognosis is the use of steroids and immunosuppressive medications. Despite the beneficial role of short-term corticosteroid treatment (dexamethasone and methylprednisolone) in managing acute COVID-19 infection, the long and persistent use of steroids in rheumatological diseases, especially at moderate to high doses (10 mg/day of prednisolone or equivalent), was associated with a higher rate of hospitalization and more severe disease [28, 29]. Patients on long-term steroids in our study showed similar outcomes. Most of our subjects (21 patients, 61.76%) were on long-term oral steroids, with doses ranging between 5 and 15 mg daily. Eight of those patients (38.1%) required hospitalization. Only 5 of 21 patients (22.73%) received prednisolone with a dose > 10mg daily, of which one patient required ICU admission. Our study showed no significant association between the severity of the COVID-19 infection and treatment with steroids, regardless of the maintenance dose.

Cytokine storm syndrome is related to the hyperactivation of T lymphocytes and increased expression of proinflammatory cytokines, such as interleukin (IL)-1, IL-6, IFN, and TNF [35]. Many studies suggested that patients maintained on IL-6 inhibitors such as tocilizumab and TNF-α inhibitors, including etanercept, adalimumab, and infliximab, were likely to have a milder disease course and a lower rate of hospital admission [9]. In our study, methotrexate was the most common maintenance medication (26.47%), followed by MMF (23.53%) and rituximab (20.59%). None of the subjects were maintained on tocilizumab. However, 5 patients (14.70%) were treated with tocilizumab after developing a severe COVID infection. All were clinically improved and discharged from the hospital, supporting evidence of the beneficial effects of tocilizumab treatment. Six patients (17.65%) were on TNF-α inhibitors. The majority of those (66.66%) had a mild infection and did not require hospitalization. Only two (33.33%) had a severe infection and required ICU admissions. Fortunately, both were discharged home in stable condition. Our data suggest no significant relationship between TNF-α inhibitors and disease severity. However, subjects on MMF or hydroxychloroquine had a significantly milder disease course than others.

Given that CD20 is important for humoral immunity, agents such as rituximab, a monoclonal antibody against CD20, had poor clinical outcomes, including increasing the vulnerability and severity of COVID-19 infection. Rituximab causes B-cell depletion and may also decrease the lymphocyte count, increasing the susceptibility to infections. A French cohort studied the relationship between the use of rituximab and the development of severe COVID infection, supporting the association of rituximab with increased ICU admission and prolonged hospital stay due to failure to develop anti-SARS-CoV-2 antibodies. Patients in the rituximab group had a higher mortality rate than those in the non-rituximab group [36–38]. In our cohort, 7 patients were on rituximab. Three out of seven patients (42.85%) on rituximab developed severe disease, had a prolonged hospital stay, and required ICU admission, but none required intubation. Three patients (42.8%) did not require hospitalization because they had a mild infection, suggesting that the relationship between rituximab and disease severity was not statistically significant in our study. Patients who developed a severe infection were treated with rituximab for 2–6 months before developing the infection. On the other hand, most patients with mild COVID-19 received their rituximab dose 7–12 months before acquiring the infection. This perhaps illustrates the importance of the median time between rituximab administration and COVID-19 infection.

The strengths of our study include being the first in the Eastern Province to evaluate the clinical characteristics of COVID-19 infection in patients with rheumatological diseases and having a majority of Saudi participants, reflecting the role of ethnicity and genetic background.

However, the study also has limitations. Firstly, the small sample size might have been influenced by the adherence to health measures such as hand washing, mask-wearing, lockdowns, and social distancing, potentially limiting the number of COVID-19 cases among patients with rheumatological diseases. Additionally, many patients in our study had milder disease courses and did not seek medical care, which could have further contributed to the smaller sample size. Secondly, our study is a single-center observational study, which may limit the generalizability of our findings to other populations and settings. Thirdly, due to the retrospective nature of the study, the data collected relied on the documentation of the primary treating team, which could introduce potential biases or inconsistencies in the data. Lastly, the study did not have sufficient data to address the duration of biological treatments and their effects on the severity of COVID-19 infection, highlighting the need for further research in this area.

## CONCLUSION

Hypertension was the most prevalent comorbidity in our study, and it was associated with more severe COVID-19 infections. Moreover, obesity, male gender, and advanced age were independent predictors of COVID-19 disease severity. Even though SLE was the most common rheumatological disease observed in our study, there was no significant relationship between SLE and the need for ICU admission. Patients treated with hydroxychloroquine and MMF for their underlying rheumatological diseases had a milder COVID-19 disease course. However, there was no significant association between the severity of the infection and the treatment with steroids (regardless of the dose) or rituximab. Medical staff must recognize the significance of the COVID-19 pattern within this population and prioritize the appropriate management of patients presenting with rheumatological diseases and COVID-19 symptoms.

## Data Availability

The analyzed datasets used in this study and all analysis output reports are available upon reasonable request from the corresponding author.
